# Collective Prediction of Individual Mobility Traces for Users with Short Data History

**DOI:** 10.1371/journal.pone.0170907

**Published:** 2017-01-30

**Authors:** Bartosz Hawelka, Izabela Sitko, Pavlos Kazakopoulos, Euro Beinat

**Affiliations:** 1 Department of Geoinformatics - Z_GIS, University of Salzburg, Salzburg, Austria; 2 CS Research Foundation, Amsterdam, The Netherlands; Universite de Namur, BELGIUM

## Abstract

We present and test a sequential learning algorithm for the prediction of human mobility that leverages large datasets of sequences to improve prediction accuracy, in particular for users with a short and non-repetitive data history such as tourists in a foreign country. The algorithm compensates for the difficulty of predicting the next location when there is limited evidence of past behavior by leveraging the availability of sequences of other users in the same system that provide redundant records of typical behavioral patterns. We test the method on a dataset of 10 million roaming mobile phone users in a European country. The average prediction accuracy is significantly higher than that of individual sequence prediction algorithms, primarily constant order Markov models derived from the user’s own data, that have been shown to achieve high accuracy in previous studies of human mobility. The proposed algorithm is generally applicable to improve any sequential prediction when there is a sufficiently rich and diverse dataset of sequences.

## Introduction

The problem of algorithmic prediction of human mobility has received significant attention in the literature in recent years, for its potential applications and its inherent theoretical value. The problem is posed as asking for a prediction of the short-term future location of an individual, given his or hers previous locations and possibly other side information. One can distinguish the algorithms that have been studied in the mobility prediction literature in two broad classes. In the first class we include algorithms such as Markov models that use only the single user’s past locations, without any other information, to estimate the next location. This individual sequence prediction is closely related to lossless compression of sequential data [1–3]. The main feature of this class of algorithms is the reliance on a single data source, which is a major practical advantage. The main disadvantage, however, is the cold start. When an agent is added to the system, a prediction algorithm utilizing only the past data of this agent to produce predictions faces the problem of having to wait enough time until a statistically significant amount of data has been accumulated, before reliable predictions can be produced. Furthermore, when the sequence of states of an agent is not stationary (the patterns of states of the agent cannot be modeled by a probability distribution function immutable in time), then a prediction algorithm based on sequentially approximating a single probability distribution will fail. This is the case for instance, when the sequences of data are short (agents are part of the system for a short amount of time, such as tourists in a foreign country). The second class comprises methods that take advantage of data beyond the user’s own past locations to improve prediction quality, mitigate the cold start and stationarity problems. This can include, for instance, prior knowledge of velocity (the user travels by car or walks) or information about the likely locations of interest for the user (e.g. the network of connections of the user’s social interactions [4–6]). The main disadvantages are of practical nature, as there are many cases in which useful secondary information is not available or is insufficient.

We study an algorithm that falls between these two classes. It uses only mobility traces to predict mobility without requiring any additional information type, but it leverages the mobility traces of other users in the same system to improve the predictability of each individual user. This approach exploits the growing availability of massive amounts of traces for the same system (for instance, GPS traces of vehicles in a city, mobile phone traces in a country, social media geospatial metadata in Twitter) to improve single-user predictions without resorting to different information types. The main idea of the method is that, given a specific user, it is likely that many other users in the past have made the same time-space transition that the user is about to make. The proposed algorithm (see section on Methods) comes from the family of sequential (online) learning prediction with experts ([7] and references therein), a family of machine learning algorithms that is well developed theoretically and has been applied to real-life prediction problems in various fields e.g. [8–20]. The algorithm (labeled EW forecaster, or EW in short, from Exponential Weights, see below) has been developed for any sequential prediction application where: 1) there is a need to minimize cold start times and predict from short sequences, 2) there is a vast availability of sequences for uses in the same system and 3) there isn’t any other type of data available to improve predictions.

In general terms, the algorithm predicts the next status of an agent in a system where the sequence of previous states of that agent are represented as time-stamped symbols from a finite set. Instead of deriving the next symbol from the agent’s sole history, the algorithm looks at the future states predicted by all other agents in the system and gives more weight to those that, in the past, best predicted the future of the agent in focus. When predicting the next location in a users’s mobility sequence, therefore, individual sequence prediction algorithms derived from other users are queried to provide a prediction for the user in focus. The advantage comes from leveraging the redundant collection of typical mobility behaviors in the area to predict individual mobility sequences. Learning and weight adaptation take place at every prediction step to minimise the prediction error.

Given the time-stamped location data of users in an area, one can construct an individual sequence prediction algorithm for each user based only on that user’s past locations: these algorithms are called *experts* and their collection an *expert ensemble*, or *expert pool*. A forecaster algorithm then uses the inputs of the experts to predict the next step of a user at each round. The initial choice of the forecaster is random, with probability proportional to the weight of each expert. After the real next location is revealed, the forecaster keeps the experts that were right and demotes the experts that were wrong. This is implemented by multiplying the weights of erroneous experts with an exponential learning factor *β* = *e*^−*η*^, where *η* > 0 is the *learning rate*, while leaving the weights of correct experts unchanged for the next round.

To build the experts from the user mobility sequences, we use an *O*(1) Markov model, constructed from the expert’s location data so that the transition probabilities between states, i.e. successive locations, are those measured in the expert’s past mobility sequence(s). Hence only a fraction of experts, those that contain relevant transitions, will be able to provide a prediction to the forecaster at any given time step. For human mobility at the scale of a country this fraction is in fact typically rather small. We apply a variant of the forecaster that accounts for the so-called sleeping experts, i.e. for situations where not all experts can make a prediction available to the forecaster at every step [13, 21–24] (*specialised* experts is an equivalent term used in the literature). In the sleeping experts version of EW, weights of sleeping experts are not updated.

In the present study we test the algorithm for predicting the next position of a users in the next hour based on anonymised mobile phone call detail records (CDR) for roamers in a European country (roamers are foreign visitors that visit the country for personal, tourism or business reasons). CDRs have been used in studies of human behavior (see e.g. [25]), and often as proxies of an individual’s location in studies of human mobility prediction [5, 26, 27]. The interest in using CDRs to study human mobility has recently received a new urge in an effort to better understand and slow down the spread of disease outbreaks [28–31].

In order for the method to provide accurate predictions using the expert/forecaster method, it is a necessary condition that the dataset has a high degree of completeness, i.e. that the users’ traces cover the area under study, possibly with redundancies. Otherwise, with a large number of instances where none or too few of the experts are able of contributing a prediction accuracy will be hit. When this basic condition is satisfied, our approach is particularly suited for predicting transient mobility behavior, without using additional data other than the time-stamped locations of users. On our test set, consisting of the thousand longest continuous mobility sequences in the seven month dataset, the EW forecaster outperforms the standard for individual sequence human mobility prediction, the Markov model [27, 32], by an average of 5% in accuracy.

The mobility of tourists and foreign visitors is naturally characterized by non-repetitive behaviors and relatively short sequences (the test set median is about one week for our dataset). Our results indicate that the EW forecaster combined with a large and diverse enough pool of mobility patterns can provide an advantage for prediction in this mobility regime.

## Results

### Test dataset

The data was provided by a major telecom operator and consists of an anonymised sample of seven months of more than 10 million roamers’ CDRs in a European country. The data set spans the period between beginning of May to end of November 2013.

Each CDR contains the principal antenna that a mobile device is connected to during a phone call, SMS communication or data connection. The time-stamped connection event is interpreted as a location measurement, positioning the device inside the approximate coverage area of the principal antenna. The size of this coverage area can range from a few tens of meters in a city to a few kilometers in remote areas. We do not consider at all the actual geographical positions of the antennas, instead we take this correspondence as a given and represent each antenna as a Unicode (utf-8) character (to accommodate for the large number of antennas in our sample). The series of connections of a roaming user is transformed into a time-stamped character sequence (*X*_1_, *X*_2_, …, *X*_*N*_), which is the object passed to the prediction algorithm.

We take the time step of the sequence of locations to be 1 hour. If more than one events fall within a single time step, one is chosen in random to represent the location of the user. In this manner, the mobility trace of a user is converted to an abstract sequence of symbols that unfolds in one-hour steps, and predictions are given for the next hour location, i.e. the next symbol in the sequence. The length of the time step is chosen to balance prediction accuracy with completeness of the sequences. The sequences of antenna connection events for most users are discontinuous and sparse, having the usual erratic profile of mobile activity patterns. A shorter time unit increases trace fragmentation, while a longer unit would reduce the accuracy of the representation of the actual mobility trace, and consequently the value of the prediction. Although many events are discarded from the original dataset when building the sequences in this manner, there is the advantage of not having to carry time information separately, as this is encoded implicitly in the position along the sequence. In an applied setup, other sampling ruled can of course be employed, without impacting the functionality of the algorithm, e.g. sequences composed of doublets of space and time positions with natural, irregular time differences between points.

The test set of sequences used for benchmarking the algorithm is selected by length, so that we can gather enough statistical data on the behavior of the algorithm. Specifically, the 1000 longest continuous character strings, i.e. mobility trace fragments, were selected. To make the comparison with individual sequence prediction sharper, we remove from the dataset the rest of the data of the users that have one or more sequences in the set of one thousand. In this way it is as if the users in the test set is observed for the first time, and past mobility patterns of the user in focus are not included in the expert pool. The remaining data is used to construct the expert ensemble. Each user’s trace is turned into a *O*(1) Markov model which then plays the role of an expert providing predictions to the forecaster whenever possible. Our sample comprises of more than 10 million users, each of which contributes a prediction algorithm to the expert ensemble. This is an unusually large number of experts. We could find a comparable expert ensemble used only in [9], in the context of document classification. This unusual abundance of experts is not incidental, instead it defines our approach. We exploit the redundancy of mobility patterns in our dataset to predict traces effectively.

We present the results of the empirical tests of the forecasters from two complementary aspects. We focus first on measuring the absolute performance of the EW forecaster on our 1000 test sequences. We then present aspects of the internal dynamics of the forecaster, and examine how the performance is affected by varying parameters that modify the content of the expert ensemble.

### Prediction accuracy

Human mobility is characterised by strong regularities that make it possible in principle to predict it with high precision [5, 26, 27, 33]. It does not however always exhibit these regularities. There are contexts in which human mobility patterns either change abruptly or are by their nature non-repetitive. Our test data of mobile network roamers exemplifies this. Tourists and foreign visitors in general are naturally expected to be less regular in their patterns than local residents, in some cases merely crossing through a country without any repetitive patterns at all. In a different sort of situation, when facing an emergency, mobility behaviors can change in a short time, usually while transitioning between more regular regimes [26]. Transient mobility behavior is also expected at the individual level as a result of changes of daily routines. Sequential learning algorithms are exactly designed to address problems where the nature of the predicted sequence cannot be accounted for a priori, or even assumed to be persistent in time. A diversified approach using many experts overseen by a forecaster is then a better strategy for accurately predicting diverse types of sequences, in our case mobility traces.

The predictive power of the forecaster/expert combination we study is drawn from the redundancies that characterise large mobility datasets. When a single user following a new, perhaps non-repetitive pattern, e.g. while visiting a foreign country for the first time, their past position data cannot inform short-term predictions. From the point of view of a dataset containing the traces a sufficiently large and diverse set of users however, patterns that are new or irregular for the individual can be found to have already been traced by other users. In the case of tourists in particular, itineraries that are a first for a user have most often already been explored by others in part or in whole. These past patterns are encoded in individual prediction algorithms and combined by a forecaster they can provide accurate predictions during a transient phase without depending on the regularities of a single individual. In addition, the expert ensemble can be dynamic, with experts added or removed on the fly, for example by choosing the experts in a moving time window of constant or varying width.

We measure the performance of the EW sleeping experts forecaster on our set of test sequences. We compare its performance with an important benchmark for human mobility prediction at this resolution and duration, the *O*(*k*) Markov model. The expert ensemble is defined (per sequence) by admitting only expert sequences that ended in a fixed time *T*_*past*_ before the predicted sequence starts. For the comparative testing we took *T*_*past*_ to be 3 months (2160 hours). We also admit all experts in our sample, without any filters. This setup provides optimal performance.

**Fig 1:**
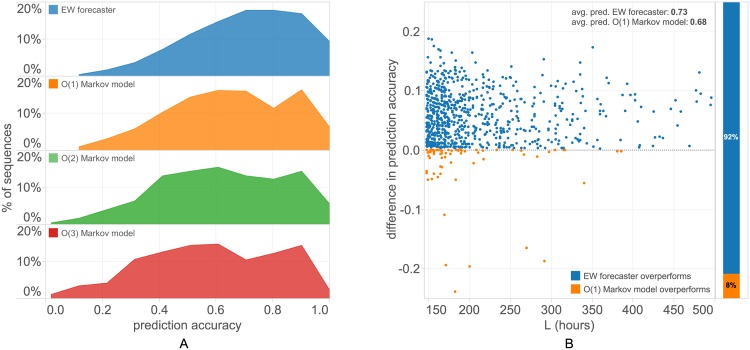
EW forecaster prediction accuracy. (A) Percentage of sequences predicted with a certain accuracy (in bins of 10%) for the EW forecaster and Markov models of order *k* = 1, 2, 3 constructed sequentially from the users own data as the sequence of locations is observed in time. We use a learning rate *η* = 3. The EW forecaster improves on the performance of the best Markov model, which again turns out to be *O*(1) [27, 32], by an average of 5%. A detailed comparison between the two is depicted in (B), the scatterplot of difference in prediction accuracy per sequence. For more than 90% of the test sequences, the EW forecaster is more accurate.

**Fig 2:**
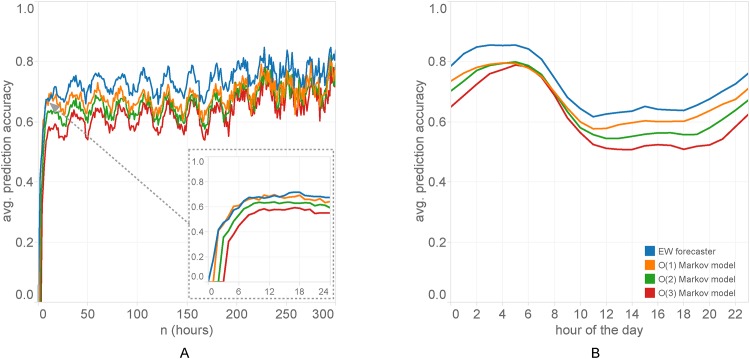
Prediction per position and over a hour of a day. (A) Average prediction accuracy per position *n* in the sequence, for the EW forecaster and Markov models orders *k* = 1, 2, 3. The best Markov model is *O*(1) and is on par with the EW forecaster for the first half-day after the start of the user’s sequence and the prediction process. EW achieves a stable (average) lead after that point. The quasi-periodic pattern is due to the fact that most roamers arrive to the visit country during the day, combined with the fluctuation between day and night prediction accuracies seen in (B). Prediction accuracy is significantly higher in the period between 02:00–08:00 because of the much higher regularity of mobility patterns during these hours.

The performance of the EW forecaster can be seen in Fig 1A, in comparison with Markov model individual sequence predictors of order *k* = 1, 2, 3. The best settings for the EW forecaster turn out to be at a value *η* ≈ 3, but the accuracy is only slightly higher than in the adaptive version (S2 Text), where the learning rate *η* is free to vary over a grid of values, and at each step is given the median of the values of *η* that have had the best performance so far. In the diagrams we always use *η* = 3. In addition, the exclusion of the user’s own location sequence (as it unfolds in time) in the expert ensemble gives a slightly better average prediction accuracy than one gets when admitting it as an additional expert. As we can see, the EW forecaster performs significantly better than Markov models, with the *O*(1) model being the most accurate among the latter. This is consistent with the results seen in previous studies of human mobility prediction. Our choice of order 1 for the Markov models of the experts is based on this ranking. The distribution of the differences in accuracy for individual test sequences shows that EW offers a 5% average advantage over the *O*(1) Markov model [Fig 1B]. When predicting a new sequence, EW overtakes the Markov models in accuracy after an average of 14 hours [Fig 2A]. The quasi-periodic pattern in [Fig 2A] is explained by the day-night variation in prediction accuracy [Fig 2B], combined with the fact that most foreign visitors arrive during the day.

### Internal dynamics of the forecaster

To understand better when and why the sequential learning algorithm works or not, we examine the internal dynamics of our forecaster/expert ensemble combinations. The sequential learning algorithm considered as a dynamical system evolving in time contains millions of degrees of freedom, namely the experts’ weights. Its dynamics are described by an equal number of difference equations that depend on the sequence under prediction, and do not avail any simple treatment. Nevertheless, empirical metrics can shed some light on the factors crucial for prediction accuracy.

**Fig 3:**
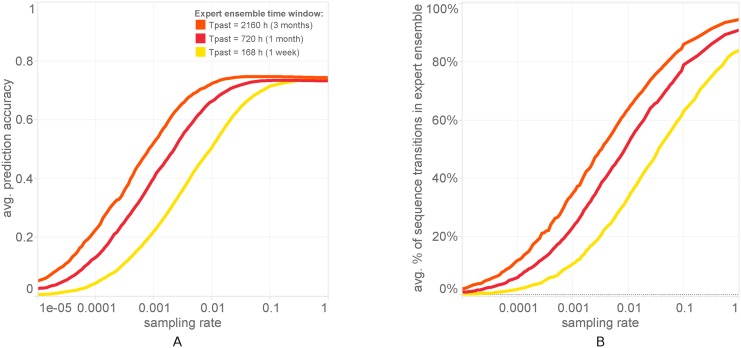
Prediction accuracy dependence on sampling and *T*_*past*_. (A) Average prediction accuracy for particular filterings of the expert ensemble. We randomly sample experts from the ensemble and additionally we filter the experts’ sequence fragments so that only those that end within a time window *T*_*past*_ are included. Decreasing the sampling rate and/or reducing *T*_*past*_ decimates the ensemble, and beyond a point it hits the accuracy of the forecaster. (B) The average percentage of distinct transitions *X*_*n*−1_ → *X*_*n*_ in a test sequence that are contained by at least one expert in the ensemble after filtering. Prediction accuracy in (A) starts dropping when the sampling rate is reduced beyond a few percent, showing that the ensemble is very diverse and robust. A very slight drop in performance comes with including all experts, due to the logarithmic search costs of the forecaster when the ensemble grows.

In Fig 3A, we see the dependence of prediction accuracy from the EW forecaster on two parameters modifying the expert ensemble, a sampling rate at which the ensemble is randomly sampled, and *T*_*past*_, the time span before the start of the predicted sequence from which prediction data is admitted to the ensemble. In both cases, performance is stable over a wide range of the parameters and quickly degrades outside. This demonstrates on one hand the robustness of the performance over changes in the expert ensemble and on the other the quick failure when the expert ensemble starts becoming incomplete. As we see, below a certain threshold of a few percent sampling rate the algorithms performance drops significantly. The fast drop in performance is due to the decimation of the transitions between locations available in the *O*(1) Markov models of the expert ensemble when experts are filtered out. In Fig 3B we plot the average percentage of unique antenna-to-antenna transitions in the test sequences which are also contained in the expert ensemble, as a function of the sampling rate. The performance of the forecaster depends most crucially on the quality of the ensemble. When the ensemble is diverse, shifting mobility patterns are quickly picked up and correctly predicted by the relevant experts. The EW forecaster significantly promotes their weights relative to other experts over just a few time steps. While the Markov model (or any individual sequence prediction algorithm) has to gather enough statistical information about the new behavior before producing correct predictions, the forecaster has most of this information already available in the experts.

**Fig 4:**
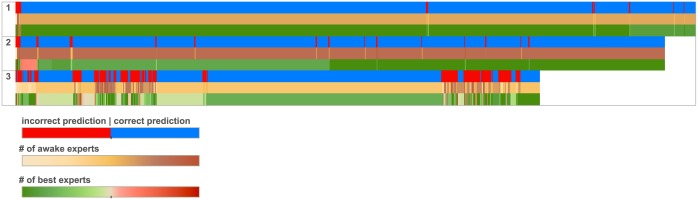
Correct/incorrect prediction for given position for three selected sequences. Prediction accuracy in our setup depends crucially on the availability of good experts in the ensemble. In the three example sequences we see a color-coded depiction of prediction success or failure adjacent to the numbers of awake and best experts, i.e. experts that can provide a prediction at a given step, and those among them which have accumulated the minimum loss up to that step. The three sequences are rather typical examples seen in the test dataset. Low numbers of best and awake experts almost invariably lead to incorrect predictions, and vice versa.

This overall dependence on the availability of transitions in the expert ensemble can be also seen when zooming in to single sequences. The three sequences shown in Fig 4 are colored in three different scales, showing the qualitative correlations between the numbers of best and awake experts and success or failure in prediction. They have been picked to represent three types of prediction dynamics typically seen in our test set. It is clear that the probability of success correlates strongly with the number of best experts at any given time step. This number can stay relatively stable over a segment and then change up or down abruptly as the user moves to a new antenna for which few experts are available. When many experts are available for the current mobility patch of the user, the forecaster quickly starts producing correct predictions.

**Fig 5:**
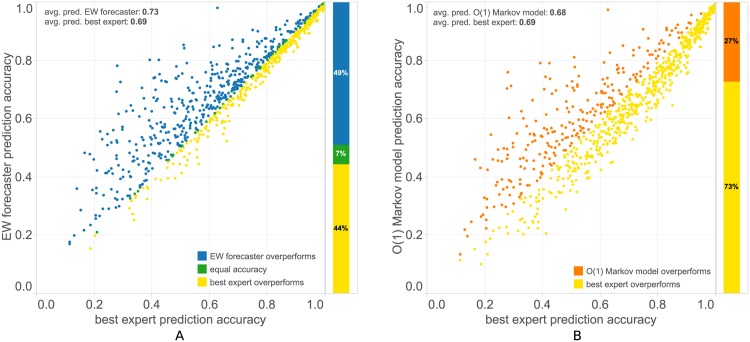
Comparison with the best expert in the ensemble. The best expert here is declared at the end of the sequence, as the Markov model in the expert ensemble which accumulated the minimum loss during prediction. If more than one experts share this property, a representative is chosen arbitrarily. (A) The EW forecaster’s prediction accuracy compared to the best expert prediction accuracy. The forecaster’s accuracy is superior more often than not, and with larger differences, resulting in a 4% average advantage. (B) The *O*(1) Markov model constructed sequentially from the user’s own locations as they are recorded in real time is less accurate than the best expert for a large majority of the test sequences. It may appear slightly surprising that another users data is better at predicting a given user’s location sequence, but the user’s own Markov model is constructed sequentially, needing time to learn the patterns, while experts’ Markov models enter the “competition” fully constructed.

The EW forecaster’s internal benchmark is *regret*, the difference in cumulative loss—here the number of erroneous predictions—between itself and the best expert, i.e. the expert with the minimum loss, in those rounds where the expert was awake [22, 23]. Here we use a different measure, comparing the forecaster’s predictive accuracy with the accuracy obtained by each expert when predicting the sequence alone. For the comparison we declare as *best expert* the one that attains the best prediction accuracy over the whole sequence. In our test set, the forecaster is often significantly more accurate than the best expert, while never performing much worse [Fig 5A]. The *O*(1) Markov model constructed sequentially from the predicted user’s data in contrast is performing more poorly, with the best expert holding a significant advantage in the majority of cases [Fig 5B]. In effect, the mobility trace of a different user is often a better predictor for another user’s trace, than the latter’s own mobility data. Of course this is due partly to the dynamical construction of the user’s own Markov model, which is populated with new transitions as they happen, while the experts’ Markov models are derived from past data in a time window *T*_*past*_ = 3 months.

## Discussion

We have shown that a large number of individual sequence prediction algorithms derived from the mobility traces of mobile phone users can be combined by an Exponential Weights forecaster to provide accurate next-hour location predictions for individual users. Using a dataset of mobility traces, we have demonstrated the potential of the method to predict short trips of transient populations such as tourists, which, in general, are non-stationary. The method can easily be implemented for CDRs, even when the data is highly incomplete, with many gaps in time. It outperforms the Markov model standard for individual sequence human mobility prediction while requiring only time stamped locations at the input.

The proposed method is domain-agnostic, and can in principle be applied to the prediction of any dataset of time series that can be encoded into character sequences. The only essential requirement is that the sequence dataset contains enough observations so that the phase space of the dynamical system under prediction is already covered, many times over if possible. This is in contrast to individual sequence prediction algorithms, where solely the data of a single sequence is needed to make predictions; it enjoys the advantage of fast adaptation to new mobility patterns for a newly observed agent, or in cases where the event sequence is transient.

## Materials and Methods

### Sequential prediction: experts and forecasters

The problem of human mobility prediction is naturally formulated as a sequential prediction problem. The sequence of positions unfolds in time, and the data available for predicting the next position lies in the past. Sequential prediction methods have been developed as a means to provide guarantees on the quality of predictions without making any a priori assumptions on the nature of the sequence. In sequential prediction with experts, the unknown character of the unfolding sequence is anticipated by combining a collection of prediction algorithms instead of a single one. The goal is to make the algorithm able to adapt to non-quasi-stationary transient patterns that may be encountered in the sequence. The benchmark for the quality of prediction is the performance of the best expert, or of an optimal combination of experts. Instead of depending only on the sequence, as in individual sequence prediction when a universal algorithm is used, the best possible prediction rate depends both on the sequence and on the ensemble of experts.

The absolute performance of an expert is measured by a loss function $l^{i}\left(Y_{n}^{i},X_{n}\right)$ that quantifies the difference between the *i*th expert’s prediction $Y_{n}^{i}$ and the actual outcome *X*_*n*_ (in our case, the user’s position in the next hour, indexed by the positive integer *n*). Among experts in a finite set, there are always one or more that will suffer the minimum loss over a given sequence. One of these experts can be chosen as representative of this class, as the best expert. The individual predictions are combined by a forecaster, an algorithm that assigns to each expert a weight *w*_*i*_ > 0 and makes a prediction *Y*_*n*_ for the actual prediction based on these weights. The forecaster’s goal is to minimise its own loss:

$$\begin{array}{c} L_{N}=\sum_{n=1}^{N}L(Y_{n},X_{n}) \end{array}$$ (1)

compared to the loss of the best expert in the ensemble, for a sequence of length *N*. This relative loss is called regret and is always measured in hindsight:

$$\begin{array}{c} R_{N}=L_{N}-\sum_{n=1}^{N}l^{b}\left(Y_{n}^{b},X_{n}\right), \end{array}$$ (2)

where *b* is the index of the best expert, i.e. the expert with the minimum cumulative loss at step *n*. Following the standard in the human mobility literature, we use the simple binary loss function

$$\begin{array}{c} l^{i}\left(Y_{n}^{i},X_{n}\right)=\delta \left(Y_{n}^{i},X_{n}\right),\;\text{where}\;\delta \left(A,B\right)=\{\begin{array}{cc} 0 & \text{if}\;A=B\\ 1 & \text{if}\;A\neq B \end{array} \end{array}$$ (3)

The forecaster loss function *L*(*Y*_*n*_, *X*_*n*_) is also taken to be *δ*(*Y*_*n*_, *X*_*n*_). An additional complication arising in mobility prediction with trace-derived experts is that at every round only a fraction of the experts can provide predictions. The expert Markov models are of fixed order (see S1 Text for a discussion on the choice of algorithms). They are constructed by counting the relative frequency of transitions between antennas in a user’s history. Users generally explore a very small subset of the possible transitions, and so only users that have in the past connected to a given antenna or a sequence of antennas can provide predictions for users currently in the same location. As a result, most experts will abstain from prediction at any given round. When experts cannot provide a prediction at every step they are called sleeping or specialised. The specific versions of the EW forecaster that we use can be found in S2 Text. For sleeping experts forecasters, theoretical bounds on regret based on varying definitions have been derived in [13, 21–24]. The bounds compare the forecaster to the best expert or convex combination of experts in those instances where the expert was awake. We do not attempt here to prove a similar bound for the variant we study, but present an empirical comparison of the EW forecaster’s accuracy of the best expert defined as the expert obtaining the highest accuracy when predicting the sequence as a single Markov model predictor outside the forecaster. The comparison shows that in fact the forecaster is never far worse than any single expert, while in a majority of cases its accuracy is higher.

The two main ingredients of an sequential learning algorithm are the mechanism for updating the weights after each round, and the rule for combining the experts. Some version of the majority vote is usually chosen for the latter. In our case the prediction outcomes are discrete characters corresponding to individual antennas. At each round the forecaster randomly picks a single prediction out of those provided by the experts with probability proportional to the weight of each expert. The weight update mechanism is a central factor behind the quality of the predictions, together with the quality of the expert ensemble. Exponential Weights forecasters penalise experts that make wrong predictions by multiplying their weights with a factor less than unity, depending on the loss and a learning rate *η* > 0. A higher learning rate accelerates the process of weight update:

$$\begin{array}{c} w_{n+1}^{i}=e^{-\eta l^{i}\left(Y_{n+1}^{i},X_{n+1}\right)}w_{n}^{i}. \end{array}$$ (4)

Experts with lowered weight will contribute less in the next round. Those that are often correct will see their predictions selected with higher probability.

We test the EW forecaster both with fixed learning rate and an adaptive version where the value of *η* is tuned sequentially in a manner similar to that used in [13]. In addition, we tested versions where the user’s own position data, encoded in an individual prediction algorithm of the same type as for the other experts, is added to the expert ensemble. These versions guarantee that in the infinite time limit the expert ensemble contains at least one expert that can reach the performance expected from an individual sequence prediction algorithm, but they consistently performed slightly worse that the corresponding variants where the user’s own Markov model was not included.

The central concept of our approach is that with enough users in the dataset, the space of mobility patterns will be densely covered. Users will show similarities in the type and frequency of transitions between antennas. When these transitions are encoded in an individual sequence prediction algorithm, e.g. a Markov model, one user’s past data can be useful in predicting another user’s future mobility. The bootstrapped predictions draw exclusively from the position data of users without making a priori assumptions about the sequence such as stationarity (S3 Text), or requiring additional data sources. The forecaster learns and adapts its choices based only on the success of each expert in providing correct predictions.

## Supporting Information

S1 Text Construction of the experts. (PDF)

S2 Text Sleeping Experts Exponential Weights forecaster. (PDF)

S3 Text Transience of test sequences. (PDF)

S1 Fig Experts fragmentation. Average length of a fragment in an expert sequence. Almost 95% of the experts have an average fragment length of 5 hours or less. The high degree of sequence fragmentation, due to inherent irregularities in the sequence of connection events in the CDR data, distorts the statistics of the user’s Markov model relative to the real frequencies of transitions. Presumably, a more complete record of transitions would increase the prediction accuracy. However, the EW forecaster benefits from the inclusion of all experts in the dataset, even those that are extremely fragmented, indicating that the completeness of the transition record is more crucial to its performance than the accuracy of the Markov models.
(PDF)

S2 Fig Eta grid and optimal Eta. (A) Average prediction accuracy as a function of *η* for a logarithmic grid of 30 points equally log-spaced between 10^−2^ and 10^3^. The average prediction accuracy reaches a peak at *η* ≈ 3 and drops only very slightly after that, even for very high learning rates. This is due to the abundance of experts in the ensemble. Severely reducing an expert’s weight after a even a single error in prediction with a large value of *η* does not hurt the accuracy because there are many similar experts in the dataset. It is unclear however if this behaviour persists for longer sequences, since there were not enough long continuous sequences in the test set. (B) Distribution of the optimal learning rate *η*, i.e. the rate that achieves the best prediction accuracy. Values of *η* are taken from a equally-spaced 30-point logarithmic grid. Most optimal *η*’s are quite large, indicating that the forecaster benefits from a strategy of immediate elimination of erroneous experts.
(PDF)

S3 Fig Entropy. Entropy per element estimate using the Lempel-Ziv estimator for a random sample of the test sequences. In an overwhelming majority of cases, the estimate has not stabilised before the sequence ends. This indicates that the mobility patterns that the sequence represents are not regular, or the sequence is too short to detect regularities.
(PDF)
