# Supplementary material for: Collective Prediction of Individual Mobility Traces for Users with Short Data History
Source: PLoS One. 2017 Jan 30;12(1):e0170907. doi: 10.1371/journal.pone.0170907 (PMC5279749; doi:10.1371/journal.pone.0170907)
Supplement: S1 Text — (PDF) [file pone.0170907.s001.pdf]

S1 Text. Construction of the experts

In general, there are no structural constraints in choosing the experts in an sequential learning setup. The guiding principle is that there be good experts in the ensemble. Bad experts, those that give many wrong predictions, are demoted and neutralised by the forecaster. The ensemble can be dynamic, with experts added or removed at will. In this study the experts are derived from the dataset of past user traces. A trace, i.e. a sequence of successive locations of the user sampled every hour, is turned into a prediction algorithm by using the relative frequencies of transitions between locations as transition probabilities in a Markov model of constant order k. If $(X_1X_2 \dots X_N)$ is one such trace, where X_n represents the location of the user at time-step i (here time steps are one hour), then an $O(k)$ Markov model can be constructed using as the transition conditional probabilities $P(X_n|X_{n-1}X_{n-2} \dots X_{n-k})$ the corresponding empirical relative frequencies of the transitions $\dots X_{n-k} \dots X_{n-2}X_{n-1} \rightarrow X_n$ as found in the sequence $(X_1X_2 \dots X_N)$. Here we use only $k = 1, 2, 3$, as these are the values that have emerged as the best choices for human mobility prediction [1, 2]. Higher values of the order, or variable order Markov models need long quasi-stationary sequences to reach their maximum performance, which in many cases can be impractical for human mobility, e.g. for the prediction of short atypical trips.

In our dataset, consisting of anonymized call detail records (CDRs) of roamers, the traces of users are in general very fragmented, because of the native irregularities in the activities (calls, SMS, data connection) contained in the CDRs, but also because of multiple visits to the country, or change of preferred roaming network during a visit. We construct one expert per user, simply compiling the statistics for the Markov model from all the available fragments. This of course leads to some distortion of the statistics compared to the real transition frequencies, which can be significant for users with very fragmented frequencies [S1 Fig.]. Complete sequences would produce more accurate experts. However, the exponential weight update mechanism ensures that the number of steps required to "discover the good experts" in the ensemble is logarithmic in the number of experts [3], and increasing the number of experts by admitting even the most fragmented actually increases the average accuracy marginally. Adding more experts to the ensemble turns out to be beneficial, because it increases the probability that good experts will be found there for a larger class of sequences, while the "search cost" for these experts scales only logarithmically.

[1] Song, L., Kotz, D., Jain, R., and Xiaoning, He. Evaluating next-cell predictors with extensive Wi-Fi mobility data. IEEE Transactions on Mobile Computing, (12), pp. 1633–1649, 2006.

[2] Lu, X., Wetter, E., Bharti, N., Tatem, A.J., and Bengtsson, L. Approaching the limit of predictability in human mobility. Scientific Reports, 3:1–9, 2013.

[3] Cesa-Bianchi, N. and Lugosi, G. Prediction, Learning and Games. Cambridge University Press, New York, NY, USA, 2006. ISBN 0521841089.
