# Supplementary material for: Collective Prediction of Individual Mobility Traces for Users with Short Data History
Source: PLoS One. 2017 Jan 30;12(1):e0170907. doi: 10.1371/journal.pone.0170907 (PMC5279749; doi:10.1371/journal.pone.0170907)
Supplement: S2 Text — (PDF) [file pone.0170907.s002.pdf]

S2 Text. Sleeping Experts Exponential Weights forecaster

Sequential learning algorithms for prediction are based on the idea of using a diversified ensemble of prediction algorithms, or experts, and combining their predictions in every round giving more weight to the predictions of algorithms that have been more accurate so far. To quantify accuracy, a loss function of two arguments, the prediction and the real outcome, is used. Each expert is assigned a weight, which is effectively decreased whenever the the expert provides a wrong prediction. The type of forecaster we use in this study is Exponential Weights, named after the mechanism it employs for updating the expert weights after each prediction round. If $l^i(Y^i_{n+1}, X_{n+1})$ denotes the cost function of the ith expert, where Y^i_{n+1} is the expert's prediction for the next sequence element at step $n-1$, and X_{n+1} the actual next element, then a wrong expert's weight is multiplied with the factor $\beta_n = e^{-\eta l^i(Y^i_{n+1}, X_{n+1})}$, where $\eta > 0$ is the learning rate at that step. There are many variants of the EW forecaster, their use depending on the constraints of the problem and the objective. In our case, experts are not always able to provide a prediction. The experts are Markov models extracted from mobility traces, which can make a prediction for another trace only when there is some overlap in locations that were visited by the users. Hence we employ the so-called sleeping (or specialised) experts variant [1–5]. At a prediction round n, if E denotes the ensemble of experts, only a subset $A_n \subseteq E$ can provide a prediction, and these are the awake experts in this round. The rest of the experts are sleeping, and their weights are not altered. The rule for combining the outcomes predicted by the awake experts into a single prediction at every round is typically some version of the majority rule. In our case it is chosen randomly among the awake experts' predictions, with the probability proportional to the weight of the expert. The procedure is summarised in the box below:

Initialize weights for each expert i: $w^i_0 = \frac{1}{|E|}$

At step $n = 1 \dots N$:

1) Collect predictions from awake experts Y^i_{n+1}, $i \in A_n$, according to the $O(k)$ Markov model transition probabilities $P_i(Y_{n+1}|X_n X_{n-1} \dots X_{n-k+1})$.

2) Pick randomly a single Y_{n+1} among the Y^i_{n+1} with probability $\frac{w^i_n}{\sum\limits_{i \in A_n} w^i_n}$.

3) Observe the true outcome and calculate the loss function value of each awake expert $l^i(Y^i_{n+1}, X_{n+1}) = \delta(Y^i_{n+1}, X_{n+1})$, $i \in A_n$.

4) Update the weights of the awake experts $w^i_{n+1} = e^{-\eta l^i(Y^i_{n+1}, X_{n+1})} w^i_n$, $i \in A_n$. Leave the weights of sleeping experts unchanged $w^i_{n+1} = w^i_n$, $i \notin A_n$.

The learning rate η is the only free parameter, and can vary adaptively along the sequence, following some additional rule that defines the adaptation. A higher learning rate means that the weights of awake experts that are wrong in their prediction are reduced more. We tested the forecaster with both constant and varying η. The mechanism of adaptation we used follows the ideas in [4]. A logarithmic grid of 30 points equally log-spaced between 10^{-2} and 10^3 was chosen for η, and at each step the forecaster used the median of the grid value that had attained the best accuracy so far. In the non-adaptive version, the same grid value was used for all sequences at every step, and the value providing the best average accuracy is chosen in hindsight. The average prediction accuracy as a function of η displays the behaviour shown in S2 Fig.(A). We see that the forecaster attains its best average performance around $\eta \approx 3$, and higher values give essentially the same result. This is a result of the very large size of the expert ensemble. Experts are typically so abundant at every step that it is a better strategy for the forecaster to effectively eliminate them from subsequent rounds even after a single erroneous prediction. The distribution of the value of the optimal learning rate η that gives the best prediction accuracy (S2 Fig.(B)) shows that almost all sequences are predicted better at high learning rates. It is not clear if this behaviour would continue when predicting very long sequences, as the median length (i.e. duration) of mobility sequences available for testing was only 182 hours.

The non-adaptive versions with $\eta > 3$ achieved the best performance of 73% average accuracy, but the adaptive versions achieved almost the same. Adaptation of η will effectively cost a few steps to the forecaster, and since the value of η beyond a certain threshold does not impact the result in our case, it does not seem to bring a benefit. However it is again unclear whether this result persists when predicting longer sequences. In addition to the adaptive/non-adaptive flavours of the forecaster, we tested changes to prediction accuracy when the Markov model of the user

that is predicted is included in the expert ensemble. To respect causality, the user's own Markov model is of course changing as the successive locations of the user unfold in time, incorporating new data in its transition probabilities. The inclusion of the user's own data surprisingly seems to lower average predictability by almost two percentage points. This is true in both the adaptive and non-adaptive versions.

[1] Blum, Avrim. Empirical support for winnow and weighted-majority algorithms: Results on a calendar scheduling domain. *Machine Learning*, 26(1):5–23, 1997.

[2] Freund, Yoav, Schapire, Robert E., Singer, Yoram, and Warmuth, Manfred K. Using and combining predictors that specialize. In *Proceedings of the Twenty-ninth Annual ACM Symposium on Theory of Computing*, STOC '97, pp. 334–343, New York, NY, USA, 1997. ACM. ISBN 0-89791-888-6.

[3] Blum, Avrim and Mansour, Yishay. From external to internal regret. In Auer, Peter and Meir, Ron (eds.), *Learning Theory*, volume 3559 of *Lecture Notes in Computer Science*, pp. 621–636. Springer Berlin Heidelberg, 2005. ISBN 978-3-540-26556-6.

[4] Devaine, M., Goude, Y., and Stoltz, G. Aggregation of sleeping predictors to forecast electricity consumption. Rapport technique, EDF R&D et Ecole normale superieure, Paris, 2009.

[5] Kleinberg, Robert, Niculescu-Mizil, Alexandru, and Sharma, Yogeshwer. Regret bounds for sleeping experts and bandits. *Machine learning*, 80(2-3):245–272, 2010.
