# Supplementary material for: Collective Prediction of Individual Mobility Traces for Users with Short Data History
Source: PLoS One. 2017 Jan 30;12(1):e0170907. doi: 10.1371/journal.pone.0170907 (PMC5279749; doi:10.1371/journal.pone.0170907)
Supplement: S3 Text — (PDF) [file pone.0170907.s003.pdf]

S3 Text. Transience of test sequences

A symbolic sequence unfolding in time is stationary when the probability for the appearance of any given subsequence at a given position does not depend on the position, in the infinite time limit. The relative frequencies of transitions $P(X_m|X_{m-1}X_{m-2}\dots X_{m-k})$, interpreted as the transition probabilities , must tend to a limit value as the length of the sequence grows, for all k. In practice, the frequency of the patterns must be stable enough in the time scale of the problem under study. A human mobility sequence can appear quasi-stationary in this sense, when observed for a few weeks or months. But over many years most individuals will travel, change place of residence or in other ways exhibit significant shifts to their mobility patterns, during what could be called periods of transient mobility. Our roamer data captures examples of such transients, when users are visiting a foreign country, outside their regular mobility regime.

To assess whether our test sequences can be considered quasi stationary we examine the standard Lempel-Ziv estimate of the entropy per element H [1–4]:

$$\lim_{N} \frac{1}{N} \sum_{n=1}^{N} \frac{\Lambda_n}{\log N} = \frac{1}{H} \tag{1}$$

Here N is the length of the sequence, and Λ_n is the length of the shortest subsequence starting at position n that has not previously been encountered in the sequence. Since H can be shown to converge to the true entropy per element of the sequence [1], which for a stationary sequence is independent of the position, a fluctuating H shows that the sequence cannot be considered quasi-stationary during the time interval of observation. Indeed, this is what we find for most sequences in our dataset, as can be seen in S3 Fig., where H is plotted as a function of the position for a random sample of sequences from the test set.

[1] Kontoyiannis, I., Algoet, P.H., Suhov, Yu.M., and Wyner, A.J. Nonparametric entropy estimation for stationary processes and random fields, with applications to english text. *Information Theory, IEEE Transactions on*, 44(3):1319–1327, May 1998.

[2] Lempel, A. and Ziv, J. On the complexity of finite sequences. *Information Theory, IEEE Transactions on*, 22(1):75–81, Jan 1976.

[3] Song, C., Qu, Z., Blumm, N., and Barabási, A-L. Limits of predictability in human mobility. *Science*, 327(5968):1018–1021, 2010.

[4] Lu, X., Wetter, E., Bharti, N., Tatem, A.J., and Bengtsson, L. Approaching the limit of predictability in human mobility. *Scientific Reports*, 3:1–9, 2013.
