# Supplementary figures and images for: Collective Prediction of Individual Mobility Traces for Users with Short Data History
Source: PLoS One. 2017 Jan 30;12(1):e0170907. doi: 10.1371/journal.pone.0170907 (PMC5279749; doi:10.1371/journal.pone.0170907)

**S1 Fig. Experts fragmentation**

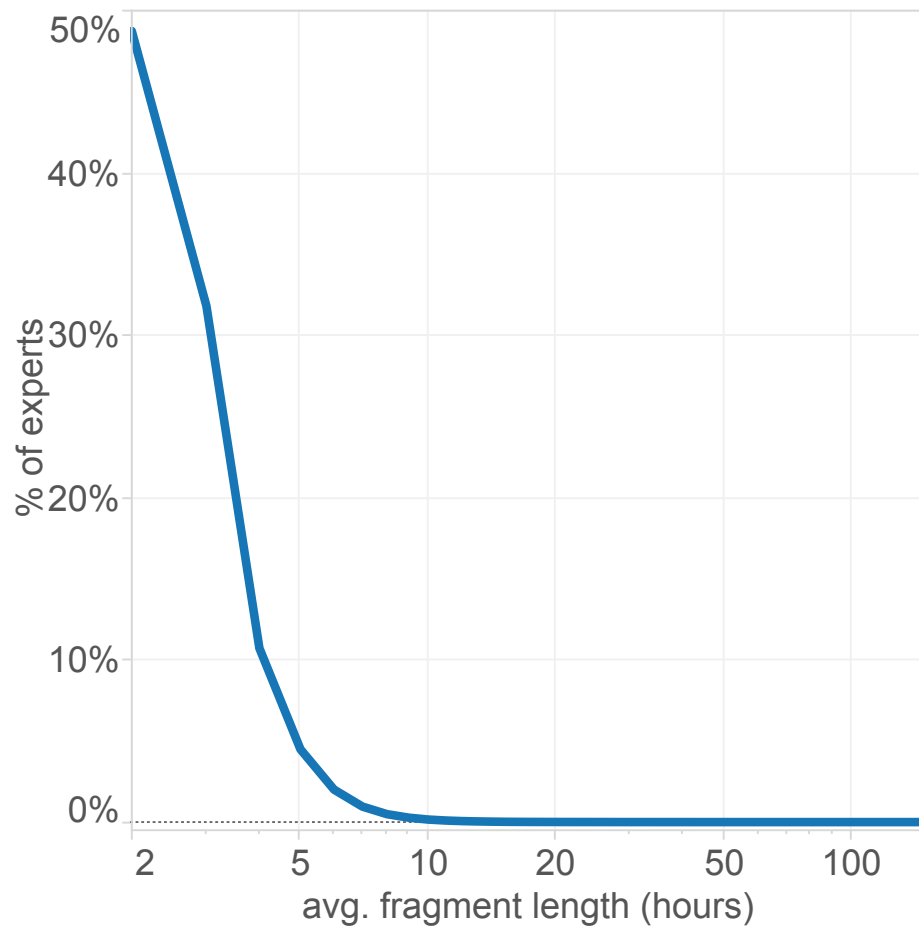

Supplement: S1 Fig — Average length of a fragment in an expert sequence. Almost 95% of the experts have an average fragment length of 5 hours or less. The high degree of sequence fragmentation, due to inherent irregularities in the sequence of connection events in the CDR data, distorts the statistics of the user’s Markov model relative to the real frequencies of transitions. Presumably, a more complete record of transitions would increase the prediction accuracy. However, the EW forecaster benefits from the inclusion of all experts in the dataset, even those that are extremely fragmented, indicating that the completeness of the transition record is more crucial to its performance than the accuracy of the Markov models. (PDF) [file pone.0170907.s004.pdf]

S2 Fig. Eta grid and optimal Eta

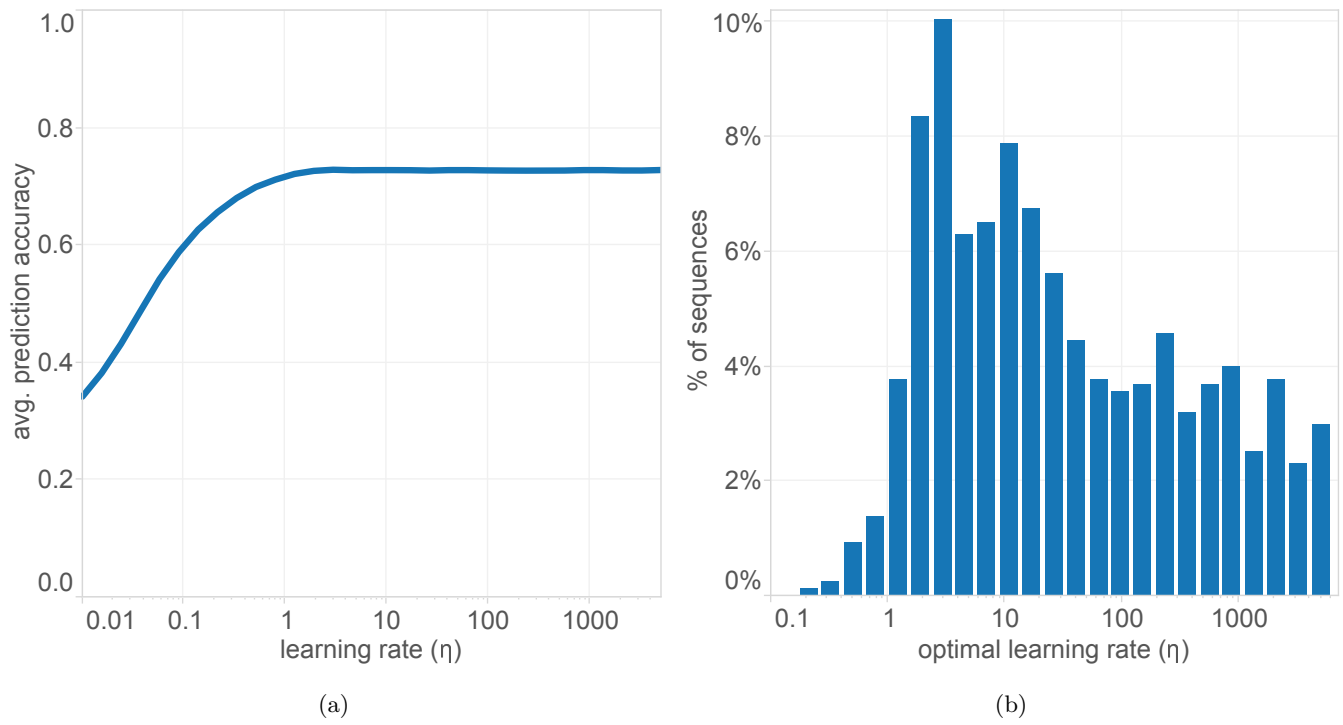

Supplement: S2 Fig — (A) Average prediction accuracy as a function of η for a logarithmic grid of 30 points equally log-spaced between 10−2 and 103. The average prediction accuracy reaches a peak at η ≈ 3 and drops only very slightly after that, even for very high learning rates. This is due to the abundance of experts in the ensemble. Severely reducing an expert’s weight after a even a single error in prediction with a large value of η does not hurt the accuracy because there are many similar experts in the dataset. It is unclear however if this behaviour persists for longer sequences, since there were not enough long continuous sequences in the test set. (B) Distribution of the optimal learning rate η, i.e. the rate that achieves the best prediction accuracy. Values of η are taken from a equally-spaced 30-point logarithmic grid. Most optimal η’s are quite large, indicating that the forecaster benefits from a strategy of immediate elimination of erroneous experts. (PDF) [file pone.0170907.s005.pdf]

**S3 Fig. Entropy**

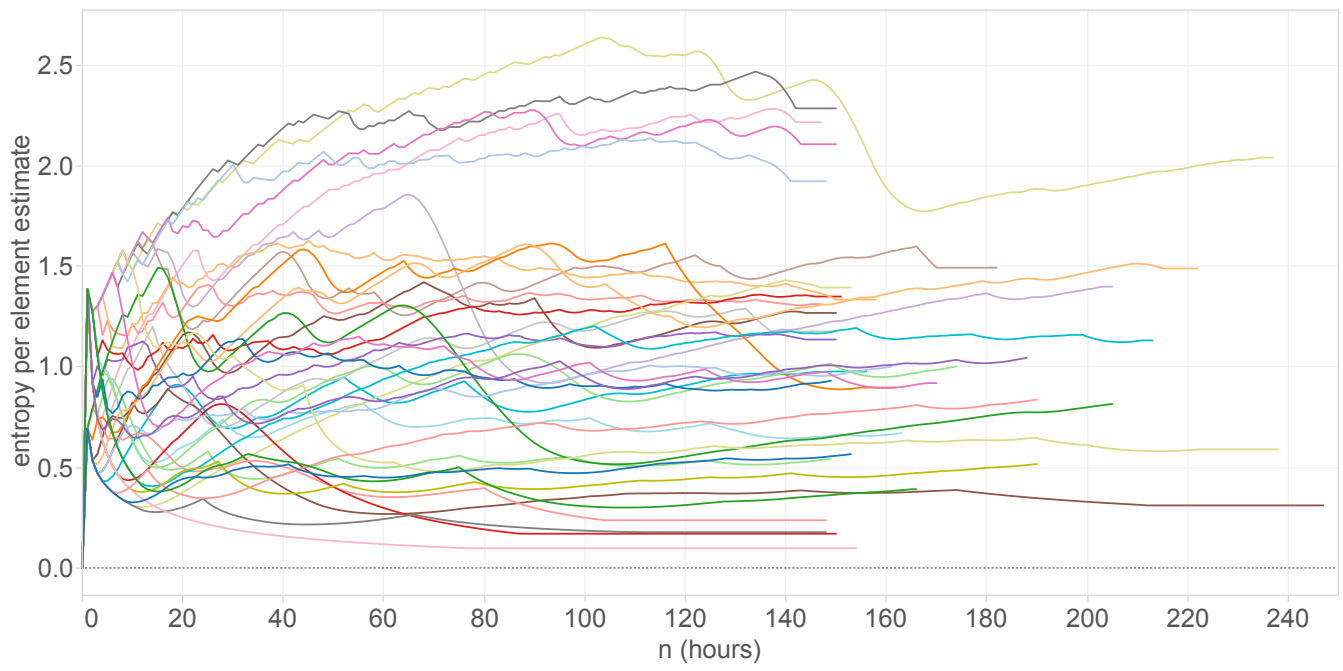

Supplement: S3 Fig — Entropy per element estimate using the Lempel-Ziv estimator for a random sample of the test sequences. In an overwhelming majority of cases, the estimate has not stabilised before the sequence ends. This indicates that the mobility patterns that the sequence represents are not regular, or the sequence is too short to detect regularities. (PDF) [file pone.0170907.s006.pdf]
